# Genome-wide identification of GAD family genes suggests *GhGAD6* functionally respond to Cd^2+^ stress in cotton

**DOI:** 10.3389/fgene.2022.965058

**Published:** 2022-09-13

**Authors:** Hui Huang, Yunxin He, Aihua Cui, Liangqing Sun, Mingge Han, Jing Wang, Cun Rui, Yuqian Lei, Xiaoyu Liu, Nan Xu, Hong Zhang, Yuexin Zhang, Yapeng Fan, Xixian Feng, Kesong Ni, Jie Jiang, Xingping Zhang, Chao Chen, Shuai Wang, Xiugui Chen, Xuke Lu, Delong Wang, Junjuan Wang, Zujun Yin, Bobokhonova Zebinisso Qaraevna, Lixue Guo, Lanjie Zhao, Wuwei Ye

**Affiliations:** ^1^ Institute of Cotton Research of Chinese Academy of Agricultural Sciences/Zhengzhou Research Base, State Key Laboratory of Cotton Biology, School of Agricultural Sciences, Zhengzhou University, Anyang, China; ^2^ Hunan Institute of Cotton Science, Changde, China; ^3^ Cotton Research Institute of Jiangxi Province, Jiujiang, China; ^4^ Department Cotton Growing, Genetics, Breeding and Seed, Tajik Agrarian University Named Shirinsho Shotemur Dushanbe, Dushanbe, Tajikistan

**Keywords:** GhGAD6, Cd^2+^, GABA, expression, evolutionary

## Abstract

Glutamate decarboxylase (GAD) mainly regulated the biosynthesis of γ-aminobutyric acid (GABA) and played an important role in plant growth and stress resistance. To explore the potential function of GAD in cotton growth, the genome-wide identification, structure, and expression analysis of GAD genes were performed in this study. There were 10, 9, 5, and 5 GAD genes identified in *G. hirsutum*, *G. barbadense*, *G. arboreum*, and *G. raimondii*, respectively. GAD was divided into four clades according to the protein motif composition, gene structure, and phylogenetic relationship. The segmental duplication was the main way of the GAD gene family evolution. Most *GhGADs* respond to abiotic stress. Clade Ⅲ GAD was induced by Cd^2+^ stress, especially *GhGAD6*, and silencing *GhGAD6* would lead to more serious Cd^2+^ poisoning in cotton. The oxidative damage caused by Cd^2+^ stress was relieved by increasing the GABA content. It was speculated that the decreased expression of *GhGAD6* reduced the content of GABA *in vivo* and caused the accumulation of ROS. This study will further expand our understanding of the relationship between the evolution and function of the GhGAD gene family and provide new genetic resources for cotton breeding under environmental stress and phytoremediation.

## Introduction

The growth of plants was affected by external environments, such as salt, alkali, drought, and heavy metal ions. Cd^2+^ was a non-essential trace metal element with significant toxicity to plants and animals, widely present in the entire soil–plant–human continuum (Zhang et al., 2021). Cd^2+^ pollution mainly came from mining, smelting, sewage irrigation, and fertilization (Qin et al., 2021). Cd^2+^ ranked first in the percentage of soil samples (7.0%) exceeding the Ministry of Environmental Protection limit in China (Shi et al., 2019). Plants absorbed Cd^2+^ through roots and accumulated *in vivo* (Chen et al., 2018). Cd^2+^ stress can inhibit plant growth through oxidative stress, inhibition of root growth, reduction of photosynthesis, disordering mineral nutrition, and water imbalance (An et al., 2019; Fu et al., 2022). Cd^2+^ accumulation in the body is mainly thought to be the enrichment of the food chain (Clemens and Ort, 2019). Therefore, remediation of heavy metal contaminated soil was extremely urgent. The application of exogenous chemicals and phytoremediation were popular methods to remediate soil heavy metal pollution (Yuan et al., 2020; Li et al., 2021).

γ-Aminobutyric acid (GABA) was a non-protein amino acid that played an important role in a variety of cellular processes. In animals, GABA was a signaling molecule that functions as an inhibitory neurotransmitter (Woo et al., 2018). In plants, the low-molecule metabolite involved in C/N balance, Krebs cycle, and as a signaling molecule, participated in plants' growth and development and response to abiotic stress (anoxia, cold, heat, drought, and Cd^2+^) and biotic stress (wounding due to herbivory and infection) (Ling et al., 2013; Scholz et al., 2015; Mekonnen et al., 2016; Ji et al., 2020). GABA can promote lipid synthesis and enhance Cd^2+^ tolerance in microalgae (Zhao et al., 2020). The study found that Glutamate (Glu)/GABA ratio was important for responding to Cd^2+^ stress. In duckweed, the application of Glu reduced the rhizoid from abscission under Cd^2+^ stress, while the opposite phenomenon occurred with the application of GABA (Yang et al., 2020).

Glutamate decarboxylase (GAD) belongs to the type II pyridoxal phosphate-dependent decarboxylase (PLP_deC) family, and catalyzes the conversion of L-glutamate to GABA irreversibly (Sandmeier et al., 1994). GAD from *Petunia hybrida* was first shown to be regulated by calmodulin (CaM) and found that only plants GAD specifically bind CaM compared to bacteria and animals (Baum et al., 1993; Baum et al., 1996). GAD activity was regulated by pH and CaM. One way was pH-dependent, plant GAD had the highest activity under acidic conditions (pH = 6), and the other way was Ca^2+^-dependent, mediated by CaM binding, which was most effective at pH 7.5 (Gut et al., 2009). GAD completed the catalytic reaction in the cytoplasm, where the pH is under the condition of alkalescence (7.4–7.5) (Gout et al., 1992). At this time, the regulation of Ca^2+^-dependent played a dominant role. When plants was under stress, such as wounds caused by pests and herbivores, and hypoxia, the cytoplasmic acidification pH will decrease (Macgregor et al., 2003; Kinnersley and Turano, 2010). Thereby inducing the activity of the GAD enzyme to enhance the synthesis of a large amount of GABA. In addition, the cytoplasmic Ca^2+^ concentration was increased and the Ca^2+^/CaM-dependent GAD enzyme activity and GABA synthesis were stimulated in response to different stress (Miyashita and Good, 2008; Kinnersley and Turano, 2010).

At present, *GAD* has been cloned in many species such as *Arabidopsis* (Miyashita and Good, 2008), rice (Akama and Takaiwa, 2007), tomato (Takayama et al., 2015), and tea (Mei et al., 2020). It was found that *GAD* was expressed in various tissues and was involved in plant growth and development (seed development, maturation, and senescence) and responded to environmental stress. *PpGAD* played an important role in hypocotyl development and vascular bundle formation (Molina-Rueda et al., 2010). *CsGAD1* was associated with citric acid utilization during fruit ripening (Liu et al., 2014). NaCl stress can inhibit the expression of *AtGAD1* and increase the expression of *AtGAD2* and *AtGAD4* (Renault et al., 2010), while hypoxia only induced the expression of *AtGAD4* (Miyashita and Good, 2008). The combined stress of high temperature and UV can significantly increase the expression of *AtGAD5*. The expression of *PgGAD* was induced under various abiotic stresses such as high temperature, hypoxia, and mechanical damage, and the PgGAD enzyme activity is enhanced under cold stress (Lee et al., 2009). *ZmGAD* responded to NaCl, drought, and low temperature (Zhuang et al., 2010), *ZmGAD1* and *ZmGAD2* can alleviate Cd^2+^ stress damage in maize by accumulating GABA (Cheng et al., 2018).

Cotton is the most widely grown commercial crop in the world. It had large biomass and a strong enrichment capacity of Cd^2+^. The main product of cotton, fiber, had less Cd^2+^ accumulation than other organs and don’t enter the food chain. Therefore, Cotton may be a potential crop to ameliorate Cd^2+^ pollution (Ma et al., 2017). However, cotton *GAD* has not yet been systematically identified and characterized. We analyzed the structure and evolution of *GAD* in cotton and aimed to provide a reference for further exploring the relationship between *GAD* and cotton Cd^2+^ stress.

## Materials and methods

### Identification of GAD family members

To obtain accurate information on the GAD family, several datasets and multiple steps were used to search for the sequences. The genome files and protein sequences of *Gossypium hirsutum* (*G. hirsutum*) (ZJU), *Gossypium barbadense* (*G. barbadense*) (ZJU), *Gossypium arboreum* (*G. arboreum*) (CRI), and *Gossypium raimondii* (*G. raimondii*) (JGI) were downloaded from the Cotton Functional Genomics Database CottonFGD (https://cottonfgd.org/) (Zhu et al., 2017). Genome data of other seven species *Arabidopsis thaliana* (*A. thaliana*), *Vitis vinifera* (*V. vinifera*), *Populus trichocarpa* (*P. trichocarpa*), *Theobroma cacao* (*T. cacao*), *Glycine max* (*G. max*), *Oryza sativa* (*O. sativa*) and *Zea mays* (*Z. mays*) were obtained from the Ensembl Plants database (http://plants.ensembl.org/i ndex.html) (Kumar et al., 2016; Wang et al., 2020). BLAST (Basic Local Alignment Search Tool) was downloaded from NCBI (https://www.ncbi.nlm.nih.gov/). The online website Softberry (http://www.softberry.com/) was used to predict subcellular localization.

A total of five published *Arabidopsis GAD* sequences as the queries. The BLAST program was used to identify all candidate cotton GAD*s* (E-value < e^−6^). The Hidden Markov Model (HMM) profile of the Pyridoxal_deC (PF01694 in Pfam) was downloaded and used in local searches of the datasets, all the possible members of the GAD gene family were retrieved using hmmer (version 3.3.1) (http://www.hmmer.org/). Then, the common id of genes obtained by the two methods was selected as the candidate genes. To further confirm these genes, these sequences were further verified via CD-Search Tool (https://www.ncbi.nlm.nih.gov/Structure/bwrpsb/bwrpsb.cgi) and Simple Modular Architecture Research Tool (SMART) (http://smart.embl-heidelberg.de/). Manually deleting sequences that do not belong to the conserved binding domain and contain incomplete C and N terminals.

The identified 10 GAD family genes sequences of upland cotton were used as probes, BLAST program was used to identify the GAD family genes in the other 7 species.

### Phylogenetic analysis and sequence alignments

The full-length amino acid sequences of 11 plant species including *G. hirsutum*, *G. barbadense*, *G. arboreum*, *G. raimondii, A. thaliana*, *V. vinifera*, *P. trichocarpa*, *T. cacao*, *G. max*, *O. sativa,* and *Z. mays* encoded by GAD genes were aligned with the ClustalW program with the default settings, and then manually adjusted in MEGA7.0. Subsequently, the neighbor-joining (NJ) tree was constructed with 1000 bootstrap replicates using the Poisson substitution (p-distance) model with default parameters in MEGA7.0 (Kumar et al., 2016). The website EvolView (https://www.evolgenius.info/evolview) was used to decorate the obtained phylogenetic tree.

### Chromosomal locations of GAD from four *Gossypium* species

The chromosomal locations of *G. hirsutum*, *G. barbadense*, *G. arboreum*, and *G. raimondii* were plotted using TBtools software (Chen et al., 2020). The reference genome GFF3 files were downloaded from CottonFGD.

### Collinearity analysis of the GAD family in four *Gossypium* species

To investigate the collinearity and to analyze the syntenic relationship among the GAD family of four cotton species, the complete genome sequences of these cotton species along with genome annotation files were subjected to the MCScanX tool (Wang et al., 2012). The collinear and homologous chromosomal regions among 4 cotton species were visualized using the advanced Circos package in TB tools. Gene duplication was assessed through MCScanX. To visualize duplicated regions in the 4 species of cotton, lines were drawn between duplicated genes in Circos using TBtools (Chen et al., 2020).

### Calculation of selection pressure

To investigate the selection pressure experienced by GAD duplicated gene pairs from 4 cotton species, the rates of synonymous (*Ks*) and non-synonymous (*Ka*) substitutions along with their ratios were calculated by *Ka*/*Ks* calculator in TBtools.

### Analysis of the conservative protein motifs and gene structure

We used the website MEME (http://meme-suite.org/tools/meme) to predict gene motifs, the parameters were as follows: the maximum number of motifs was 15, and the rest parameters were set by default (Bailey et al., 2009). The file of the structure domain was obtained from the CD-Search Tool. The software TBtools was used to draw the association analysis diagram of the evolutionary relationship, gene structure, domain, and motifs composition of genes.

### Analysis of *GhGADs* promoter regions and different expressions

The 2000 bp DNA sequence of the upstream region of GhGADs was obtained from the CottonFGD database (http://www.cottonfgd.org/) (Zhu et al., 2017). The predicted *cis*-acting elements related to abiotic stresses and plant hormones in promotor regions of the GhGADs were obtained from the PlantCARE website (http://bioinformatics.psb.ugent.be/webtools/plantcare/html/) for further analysis. We used RNA-Seq data (PRJNA490626) from NCBI (National Center for Biotechnology Information) (https://www.ncbi.nlm.nih.gov/) to analyze the expression level (FPKM) of GhGADs under cold (4°C), heat (37°C), salt (0.4 M NaCl), and PEG (200 g/L) stress (Hu et al., 2019). RNA-Seq data (GSE126671) (Han et al., 2019) from NCBI to analyze the FPKM of GhGADs in different tissues under 4 mM Cd^2+^ treatment for 9 h. The heatmaps were drawn based on the FPKM of GhGADs. Finally, TBtools software was used to draw a picture containing an evolutionary tree, *cis*-acting elements, and a heatmap of expression levels for visual observation.

### Gene interaction network of the GhGAD6 proteins

The GhGAD protein interaction network was analyzed through the STRING database (https://string-db.org/) (Zhang et al., 2022). On the basis of *A. thaliana* orthologs to predict the interaction of GhGAD family genes with other genes in cotton.

### Cd^2+^ treatment and quantitative real-time (qRT-PCR) analysis

Han 242 was used as a material with better Cd^2+^ tolerance (Han et al., 2019). We planted Han 242 in the sand, The cultural conditions were 28°C/16 h of light and 25°C/8 h of dark cycle culture. Cotton plants were soaked in 4 mM Cd^2+^ solution at the three-leaf-one-heart stage, take the root at various periods (0, 3, 6, 9, 12, 15 h) and place in liquid nitrogen (Wang et al., 2021).

We extracted RNA and reverse transcription into cDNA as a template for qRT-PCR. The primers for qRT-PCR of GhGADs are designed on NCBI (Supplementary Table S1). According to the manufacturers protocol using TransStart Top Green qPCR Supermix (TransGene Biotech Co., LTD, Beijing, China), the qRT-PCR experiment was performed on the Bio-Rad 7500 fast fluorescence quantitative PCR platform and the experiment was carried out in three independent replicates. Actin (AY305733) was used as an internal reference gene, and the relative expression level of GhGADs was calculated using 2^−ΔΔCt^, then the significance analysis was carried out in SPSS software.

### Vector construction and virus-induced gene silencing (VIGS) experiment

To verify the function of the GAD genes, we selected a highly expressed gene *GhGAD6* (*GH_D01G1621*). The fragments of 300 bp were designed by SGN-VIGS (https://vigs.solgenomics.net/). The fragment was ligated into the pYL156 vector. The recombinant vector was transformed into Agrobacterium tumefaciens GV3101. We injected GV3101 bacterial solution carrying control pYL156 (empty vector), pYL156:*GhGAD6*, pYL156:*PDS* (positive control), and pYL192 (helper vector) into the cotyledons of Han 242. After 24 h of dark treatment, cotton was grown in an incubator with 25°C/16 h of light and 23°C/8 h of dark cycle culture (Fan et al., 2022). Subsequent treatment with Cd^2+^ is described in 2.9.

### Measurement of chlorophylⅡ content and the SOD activity

The SPAD-502 PLUS measuring instrument was used to detect the chlorophylⅡ content in the leaves (Konica Minolta (China) Investment Ltd). After Cd^2+^ treatment, 0.1 g of sample powder mixed with at least 20 cotton plants were taken to determine the superoxide dismutase (SOD) activity by the SOD activity detection kit (Nanjing Jiancheng Bioengineering Institute, A001-3-1).

### Histochemical detection of H_2_O_2_

H_2_O_2_ was detected by diaminobenzidine (DAB) staining as described previously (Ji et al., 2020). Two leaves per plant were taken from three randomly selected plants of pYL156 and pYL156:*GhGAD6* lines under Cd^2+^ stress. The leaves were placed in 1 g/L DAB staining solution and treated in the dark at 28°C for 12 h, then add 95% ethanol to decolor the leaves. A deep brown polymerization product represented the reaction between DAB and H_2_O_2_.

## Results

### Identification of GAD family members

29 sequences were retrieved from 4 *Gosspium*, 10, 9, 5, and 5 putative GAD proteins and were detected by genome-wide identification analysis in *G. hirsutum*, *G. barbadense*, *G. arboreum,* and *G. raimondii*, respectively. The open reading frame (ORF) of all 10 GhGADs ranges from 1437 (*GhGAD3*) to 1512 (*GhGAD8*) bp. The encoded proteins range from 478 (*GhGAD3*) to 503 (*GhGAD8*) amino acids, with pI varying from 5.747 (*GhGAD4*) to 7.14 (*GhGAD5*) and MWs varying from 54.229 (*GhGAD3*) to 57.323 (*GhGAD8*) kDa. Their exons are 5 or 6 (Supplementary Table S2).

Select *T. cacao* that is closely related to *Gossypium*, and *A. thaliana*, *O. sativa*, *Z. mays*, *V. vinifera*, *G. max,* and *P. trichocarpa* that are more studied in plants. GAD family genes were identified in 7 other species, 5 in *A. thaliana*, 5 in *O. sativa*, 5 in *Z. mays*, 4 in *V. vinifera*, 8 in *G. max*, 2 in *T. cacao*, and 9 in *P. trichocarpa*. Then we renamed the GADs based on their location on their chromosome (Supplementary Table S3). The two tetraploid cotton species *G. hirsutum* and *G. barbadense* had twice the number of GAD family genes as the two diploid *G. arboreum* and *G. raimondii*. The GAD family genes of tetraploid cotton are significantly more than other species, indicating that cotton had undergone a large-scale expansion during its evolution.

### Phylogenetic analysis of GAD

**FIGURE 1:**
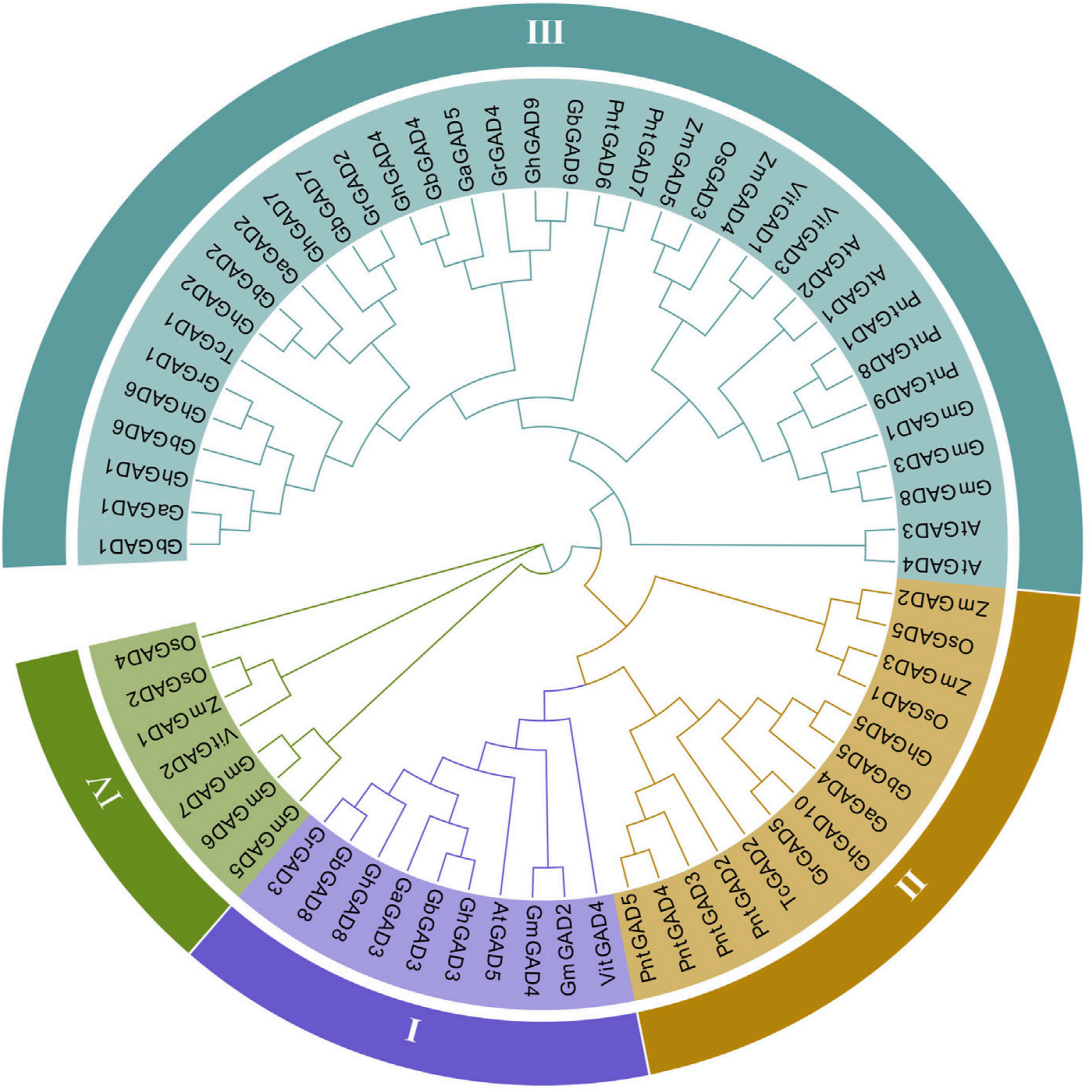
Phylogeny tree constructed using MEGA7 by the Neighbor-Joining (NJ) method. Phylogenetic relationship of the 67 identified *GAD*s from *G. hirsutum*, *G. barbadense*, *G. arboreum*, *G. raimondii*, *A. thaliana*, *V. vinifera*, *P. trichocarpa*, *T. cacao*, *G. max*, *O. sativa,* and *Z. mays*. The tree shows 4 major phylogenetic subfamilies.

To understand the evolutionary relationship of the GAD family, we utilized 67 protein sequences to build a phylogenetic tree from *G. hirsutum*, *G. barbadense*, *G. arboreum*, *G. raimondii*, *A. thaliana*, *V. vinifera*, *P. trichocarpa*, *T. cacao*, *G. max*, *O. sativa,* and *Z. mays* (Figure 1). GADs can be divided into four clades based on sequence similarity, tree topology, gene structural characteristics, and motifs in each sequence (Figure 3). The results showed that the GADs clade Ⅲ had the largest number (36), of which 6 were *GhGAD*, clade Ⅰ and clade Ⅱ had 10 and 14 genes, respectively, each containing 2 GhGAD family genes. Clade Ⅳ contains 7 genes, namely *GmGAD5*, *GmGAD6*, *GmGAD7*, *VitGAD2*, *ZmGAD1*, *OsGAD2*, and *OsGAD4*, which were less close to the genes in clade Ⅰ, Ⅱ, Ⅲ. *AtGAD* only exists in clade Ⅰ, Ⅲ, indicating that GADs in clade Ⅱ have different functions. *CcGAD* is close to the cotton GAD branch, which showed cocoa and cotton are closely related and originated from the same ancestor, consistent with previous studies (Li et al., 2014). *T. cacao* contains only two GAD*s*, revealing that the important role of GAD*s* in evolution has been amplified.

### Chromosomal location analysis of the GAD family

**FIGURE 2:**
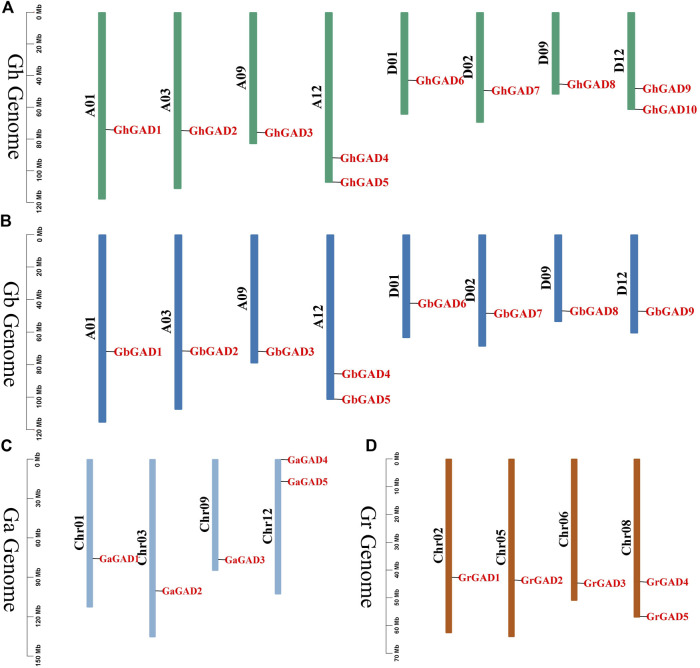
Chromosome distribution of GAD gene family in four *Gossypium*. **(A)** Chromosomal location of GADs on chromosomes in *G. hirsutum*. **(B)** Chromosomal location of GADs on chromosomes in *G. arboreum*. **(C)** Chromosomal location of GADs on chromosomes in *G. barbadense*. **(D)** Chromosomal location of GADs on chromosomes in *G. raimondii*. The scale of the genome size was given on the left.

To gain a more intuitive understanding of the distribution of genes on chromosomes, we constructed physical maps of the chromosome distributions of GAD gene family members in four cotton species (Figure 2). Chromosome location analysis showed that the chromosomal locations were unevenly distributed, GADs of *G. hirsutum* and *G. barbadense* were distributed on chromosomes 1, 3, 9, and 12 of At and chromosomes 1, 2, 9, and 12 of Dt, respectively (Table 1). The chromosome distribution of GADs in *G. arboreum* was consistent with the At, but the locations were different. The distribution on the chromosome of *G. raimondii* was different from the Dt, which implied the GADs rearrangement occurred in the process of tetraploid. A *GAD* gene is missing at the end of chromosome 12 in Dt of *G. barbadense*, compared with that of *G. hirsutum*. We supposed that may be due to the loss of the *G. barbadense* gene during evolution or incomplete genome assembly.

**TABLE 1:** Comparison of the chromosome harboring number of GADs from different genomes and subgenomes of four *Gossypium* (Ga, Gr, Gh, and Gb).

Chr. no.	Ga	Gh-At	Gb-At	Gr	Gh-At	Gb-Dt
Chr. 1	1	1	1	0	1	1
Chr. 2	0	0	0	1	1	1
Chr. 3	1	1	1	0	0	0
Chr. 5	0	0	0	1	0	0
Chr. 6	0	0	0	1	0	0
Chr. 8	0	0	0	2	0	0
Chr. 9	1	1	1	0	1	1
Chr. 12	2	2	2	0	2	1
Total	5	5	5	5	5	4

### Motifs and domain architecture and exon–intron structure analysis of GAD

**FIGURE 3:**
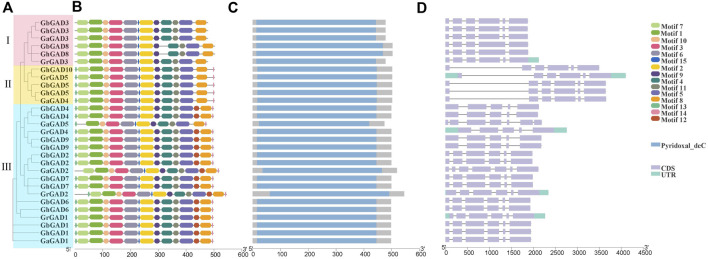
Conservative motifs, domain, and exon-intron organization of GAD gene family from *G. hirsutum*, *G. barbadense*, *G. raimondii,* and *G. arboreum*. **(A)** Phylogenetic tree of GAD gene family obtained according to NJ method in MEGA software. **(B)** Conservative motifs of GAD proteins. The motif information was obtained from the MEME webpage and visualized in TBtools. **(C)** Conservative domain of GAD proteins. **(D)** Exon–intron structures of GAD gene family.

We analyzed evolutionary relationships, motifs, domains, exons, and introns to study the conserved structure of GAD family genes (Figure 3). All GAD family genes had Pyridoxal_deC (Type II pyridoxal phosphate-dependent decarboxylase) domain, which can bind to PLP to achieve its catalytic function (Figure 3C).

They were classified according to the tree topology of the evolutionary tree (Figure 3A). The distribution patterns of exons and introns correlate with their biological functions, and their arrangement can be used to analyze evolutionary associations between members of different gene families. Interestingly, the first introns of GAD*s* in clade Ⅱ (*GhGAD5* and *GhGAD10*) were significantly longer than the others (Figure 3D). GAD family gene motifs were relatively consistent. *GADs* in clade Ⅰ (*GhGAD3* and *GhGAD8*) lacked motif 13 at the N-terminus and motifs 12 and 14 at the C-terminus. GAD*s* in clade Ⅱ lacked motif 12 at the C-terminus (Figure 3B). It was speculated that functional changes had occurred during evolution.

### Gene duplication and collinearity analysis

**FIGURE 4:**
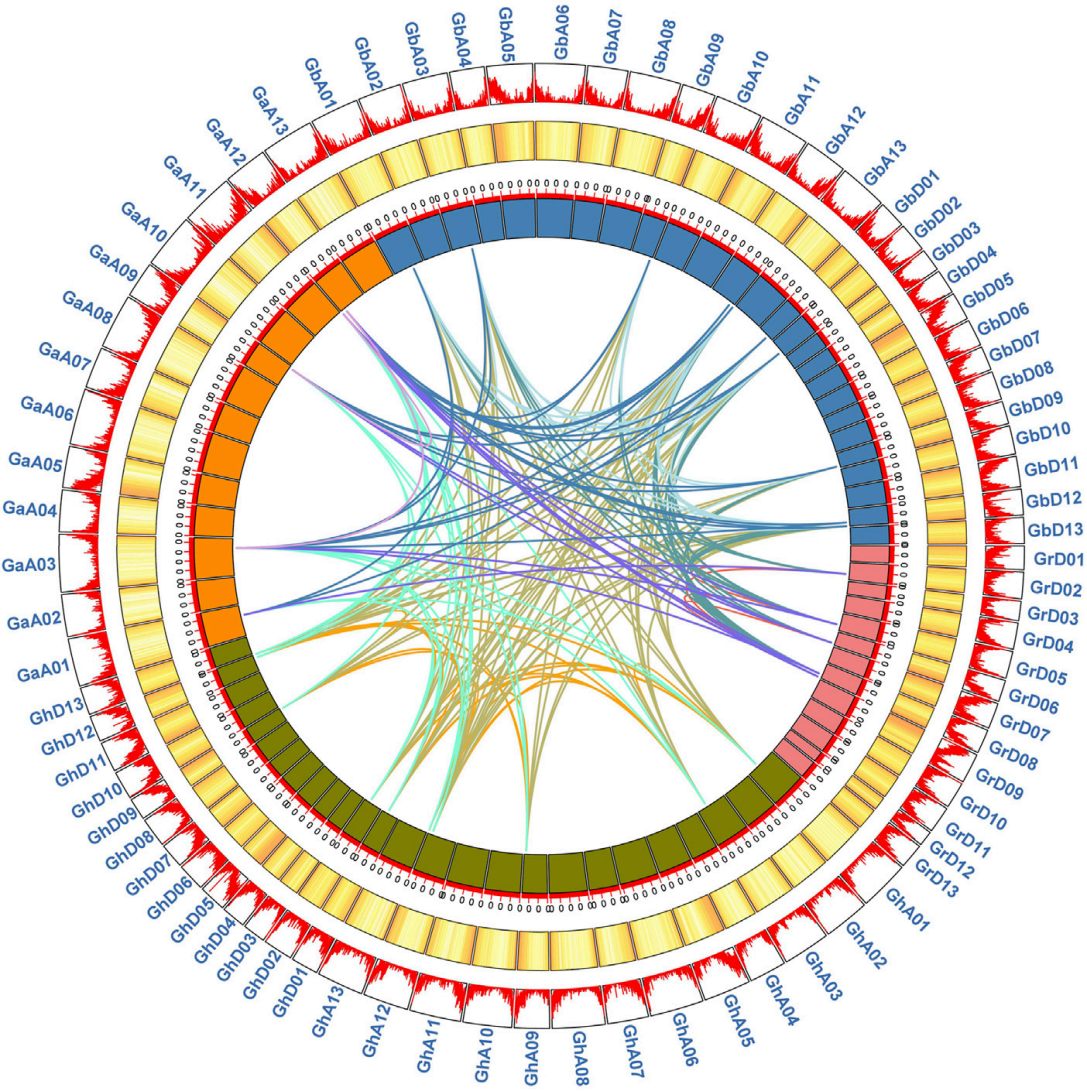
Syntenic relationship of duplicated genes pairs of GADs from four *Gossypium* (*G. hirsutum*, *G. barbadense*, *G. arboreum,* and *G. raimondii*). Chromosomal lines represented by various colors indicate the syntenic regions around the GADs. The heatmap and line map of the outer ring represents the density of genes on chromosomes.

Gene duplication events are considered to play an important role in the amplification of gene families. To explore the amplification mechanism of the GAD gene family, by comparing the genomes of Ga-Ga, Ga-Gb, Ga-Gh, Gb-Gb, Gb-Gr, Gb-Gh, Gr-Gr, Gr-Ga, and Gh-Gh, a total of 114 homologous gene pairs were identified. There were 21, 21, 2, and 4 duplication GAD gene pairs identified in Gh-Gh, Gb-Gb, Ga-Ga, and Gr-Gr, these 48 paralogous gene pairs were predicted as segmental duplications according to the chromosomal location (Figure 4). There were 66 GAD gene pairs that underwent whole genome duplication (WGD), the numbers were 21, 22, and 23 in Ga-Gb, Ga-Gh, and Gb-Gr, respectively. From these results, we presumed that segmental duplication and WGD were the main reason for the evolution of the GAD gene from diploid to tetraploid.

### Calculation of selection pressure (*Ka*/*Ks*) during evolution

**FIGURE 5:**
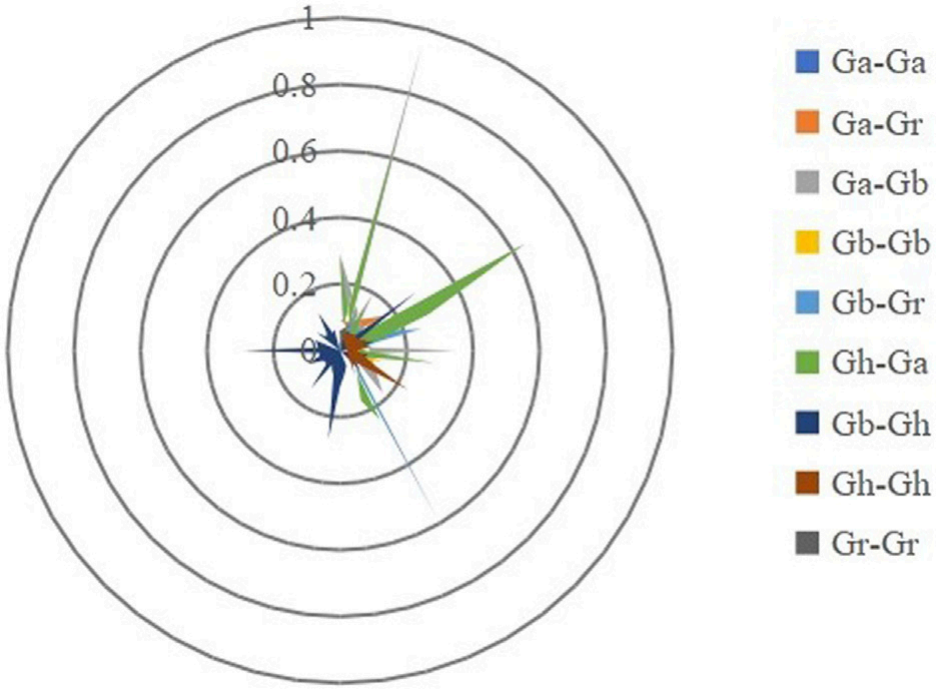
Prediction of a number of duplicated gene pairs involved in different combinations from four Gossypium species. Gh represents *G. hirsutum*, Gb represents *G. barbadense*, Ga represents *G. arboreum,* Gr represents *G. raimondii*. Different colors represent *Ka*/*Ks* gene pairs between Gb-Gb, Gr-Ga, Gr-Gr, Ga–Ga, Gh-Gb, Gh-Gh, Gh-Gr, Gb-Gr, Gb-Ga.

During evolution, duplicated gene pairs may also deviate from their original functions, eventually leading to neofunctionalization (loss of original function), subfunctionalization (a division of original function), and neofunctionalization (gain of new function). To investigate the driving forces of the GAD family gene during evolution, we calculated non-synonymous substitution (*Ka*) and synonymous substitution (*Ks*) values for 164 repeated gene pairs from four *Gossypium* (Figure 5). The selection pressure of duplicate gene pairs can be inferred according to the ratio of *Ka*/*Ks*. It is generally believed that *Ka*/*Ks* = 1 indicates neutral selection (pseudogene), *Ka*/*Ks* < 1 indicates purification or negative selection (purification selection), and *Ka*/*Ks* > 1 indicates positive selection. There are 164 duplicate gene pairs in the GAD family genes in the four *Gossypium*, including Ga-Ga, Ga-Gb, Ga-Gr, Gb-Gb, Gb-Gr, Gh-Ga, Gh-Gb, Gh-Gh, Gh-Gr, and Gr-Gr. 153 (97%) duplicate gene pairs *Ka*/*Ks* ratio are between < 0.5, 5 (3%) duplicate gene pairs *Ka*/*Ks* ratio are between 0.5 and 0.99, they are *GaGAD2*-*GbGAD2*, *GaGAD2*-*GhGAD2*, *GaGAD5*-*GbGAD4*, *GaGAD5*-*GhGAD4*, *GrGAD4*-*GbGAD9*, indicating that GAD is evolving slowly and had strong purifying selection pressure with the limited functional divergence that occurred after segmental duplications and WGD (Table 2).

**TABLE 2:** Prediction of the number of duplicated gene pairs involved in different combinations from four *Gossypium*.

Pairs	Positive	Pure selection		Total
		0.5–0.99	0–0.49	
Gh-Gh	0	0	21	21
Gh-Gb	0	0	48	48
Gh-Ga	0	2	20	22
Gh-Gr	0	0	0	0
Gb-Ga	0	2	18	20
Gb-Gr	0	1	21	22
Gb-Gb	0	0	18	18
Gr-Gr	0	0	4	4
Gr-Ga	0	0	9	9
Ga-Ga	0	0	2	2
Total	0	5	161	166
Purity%	0.00	3.01	96.99	100.00

### Promoter and expression analysis under stress conditions of *GhGAD*

**FIGURE 6:**
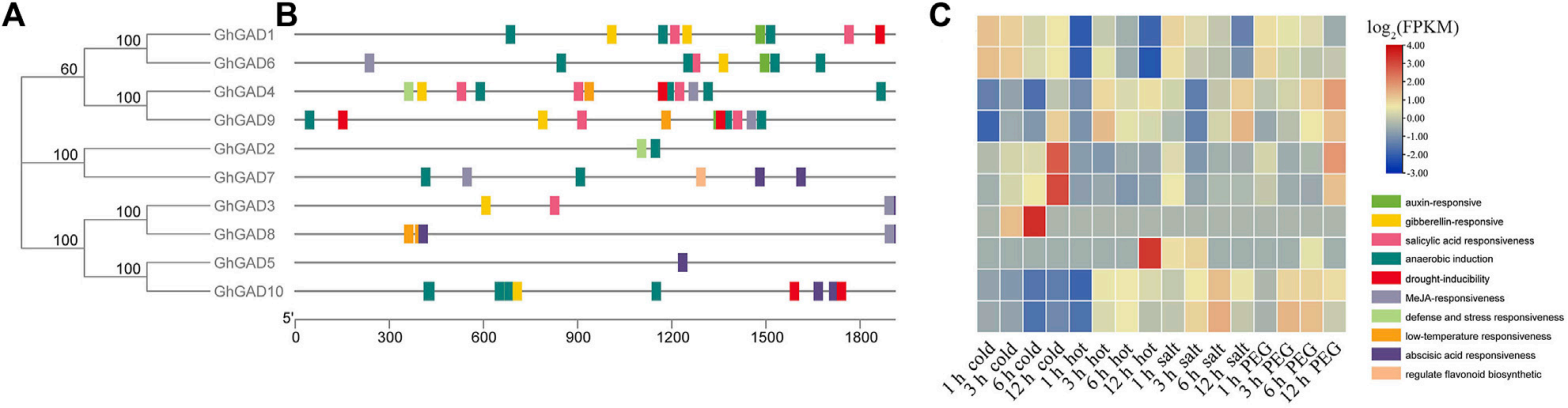
Analysis of promoters and differentially expressed GhGAD gene family. **(A)** Phylogenetic tree of GhGAD gene family. **(B)** *Cis*-elements in promoters of GhGAD gene family. **(C)** Differentially expression levels of GhGAD gene family under cold, hot, salt, and PEG stress.

Through promoter analysis, we understand the response of GhGAD to hormonal and abiotic stress, which was conducive to further analysis of the regulatory network. GhGAD is related to plant hormones (ABA, MeJA, GA, IAA, SA) and various stresses (low temperature, drought, hypoxia, defense, and stress responsiveness) (Figure 6B, Supplementary Table S4). *GhGAD10* contains an MYB binding site that regulates flavonoid synthesis. GAD contains many GA and MeJA regulatory elements. *GhGAD5* and *GhGAD10* increased at 1–6 h and decreased at 12 h after salt, heat, and drought treatments, which may be involved in the regulation of salt, heat, and drought (Figure 6C). *GhGAD2* and *GhGAD7* were significantly up-regulated after cold stress, and *GhGAD4* and *GhGAD9* were up-regulated by PEG induction.

### Tissue-specific expression of GhGAD

**FIGURE 7:**
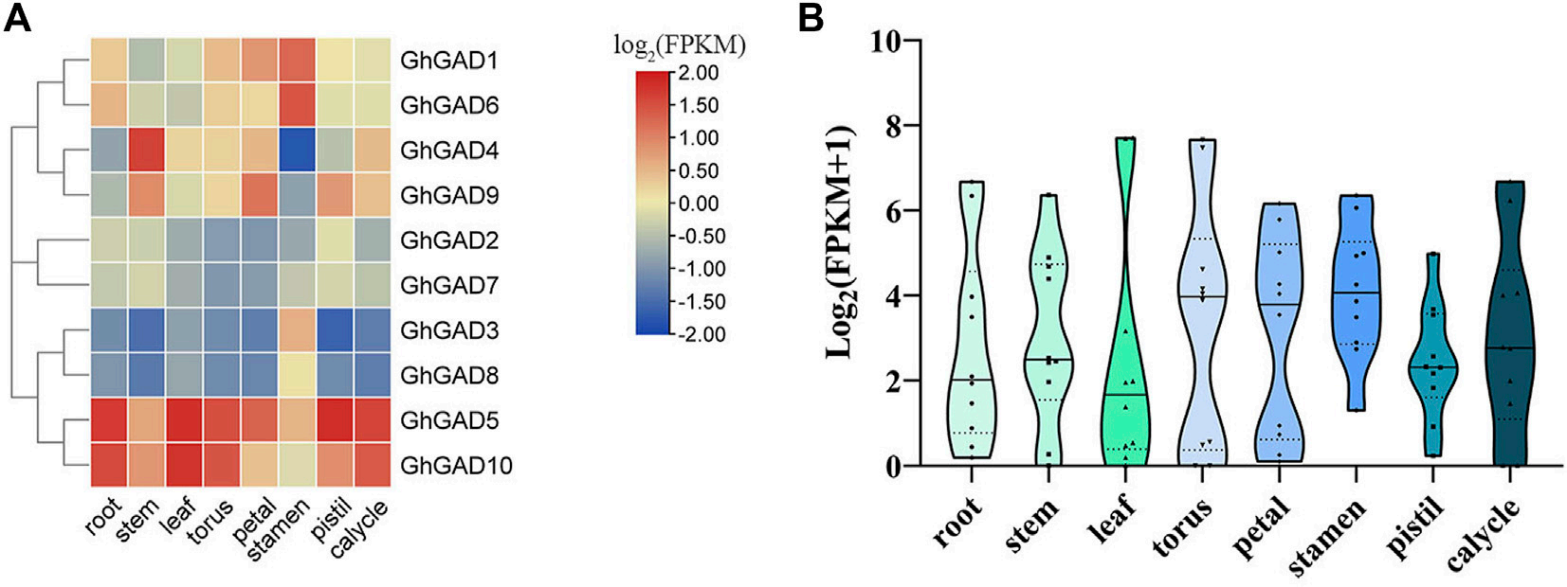
*GhGAD* genes display tissue-differential expression under normal conditions. **(A)** The color represents the gene expression values of RPKM of GhGAD genes transformed by log_2_. **(B)** The expression distribution of all *GhGADs* in each tissue.

To analyze the expression patterns of the GAD family in different tissues of cotton, we used transcriptome data to analyze the FPKM values of cotton 8 tissues (roots, stems, leaves, torus, petal, stamen, pistil, and calycle). The result showed that GADs were expressed in various tissues, and the overall expression is the highest in stamens (Figure 7). *GhGAD5* and *GhGAD10* were highly expressed in all tissues, and their expression was significantly higher than that of other GAD family genes, which may be necessary to maintain the normal life activities of cotton. *GhGAD3* and *GhGAD8* were specifically expressed in stamens, we speculate that they may play an important role in the development of stamens. *GhGAD4* was highly expressed in stems, *GhGAD9* was highly expressed in petals, and GAD orthologous gene pairs showed different expression patterns. GAD gene family is highly expressed in stamens (Figure 7B). These results suggest that GAD had tissue-specific expression under normal growth conditions.

### Expression pattern of GhGADs under Cd^2+^ stress

**FIGURE 8:**
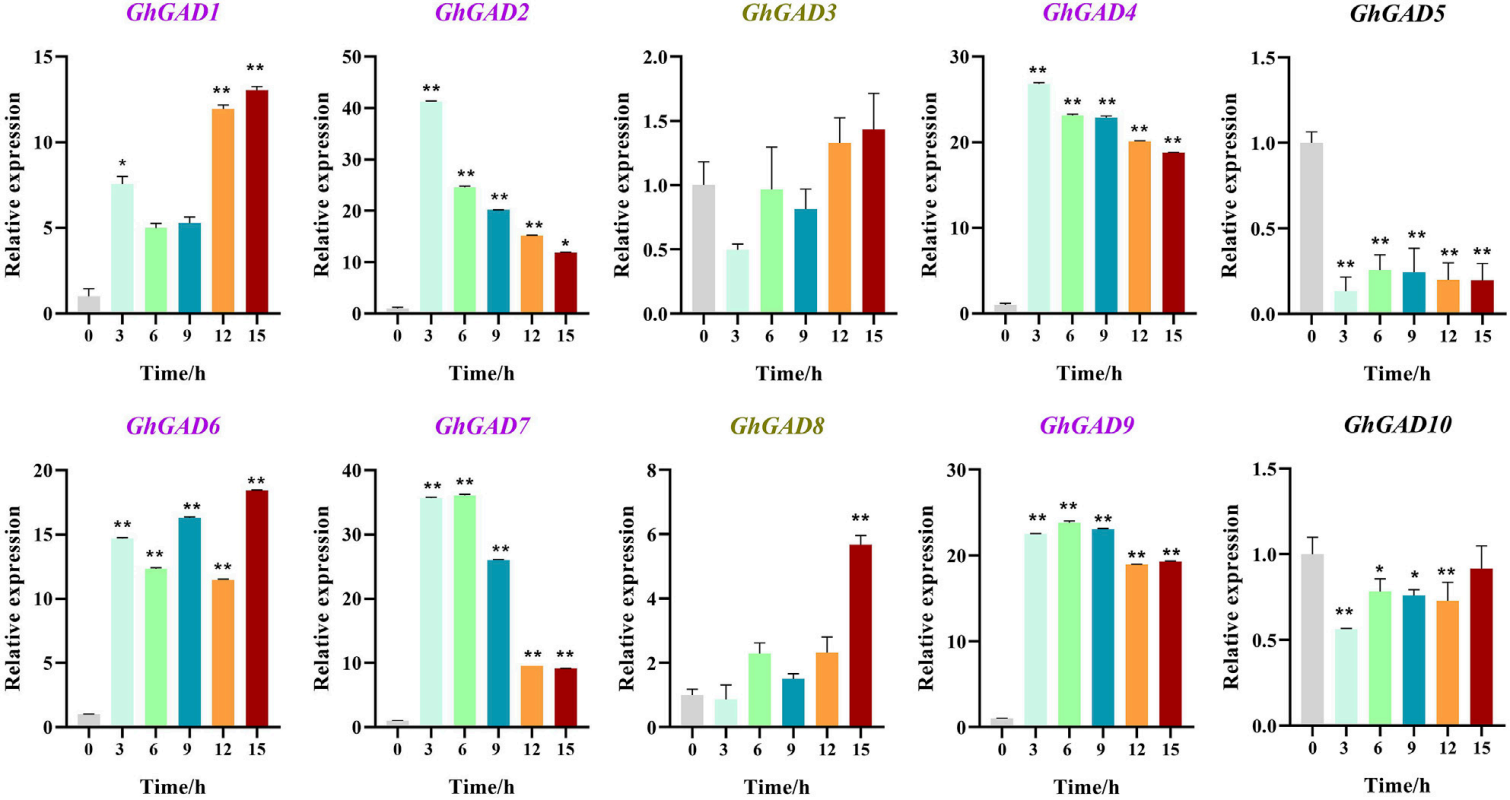
Expression analysis of GhGAD gene family at different Cd^2+^ treatment times. The bar graphs represent the relative expression levels of GAD family genes in roots under Cd^2+^ stress for 0, 3, 6, 9, 12, and 15 h (*0.01 < *p* < 0.05, **p < 0.01); the resulting mean values were presented as relative units. Error bar represents SD.

To investigate GhGADs responses to abiotic stress, especially Cd^2+^, we used qRT-PCR to study the expression changes of the GAD gene in upland cotton roots under Cd^2+^ (Figure 8). The expression levels of *GhGADs* in clade Ⅲ (*GhGAD1*, *GhGAD2*, *GhGAD4*, *GhGAD6*, *GhGAD7*, and *GhGAD9*) increased significantly after 3 h under Cd^2+^ stress and continued high expression thereafter. The clade Ⅱ GhGAD genes (*GhGAD5* and *GhGAD10*) decreased significantly after stress. The clade Ⅰ GhGAD genes (*GhGAD3* and *GhGAD8*) did not change significantly. The results revealed that different clades of GAD family genes had different expression patterns in response to Cd^2+^ stress.

**FIGURE 9:**
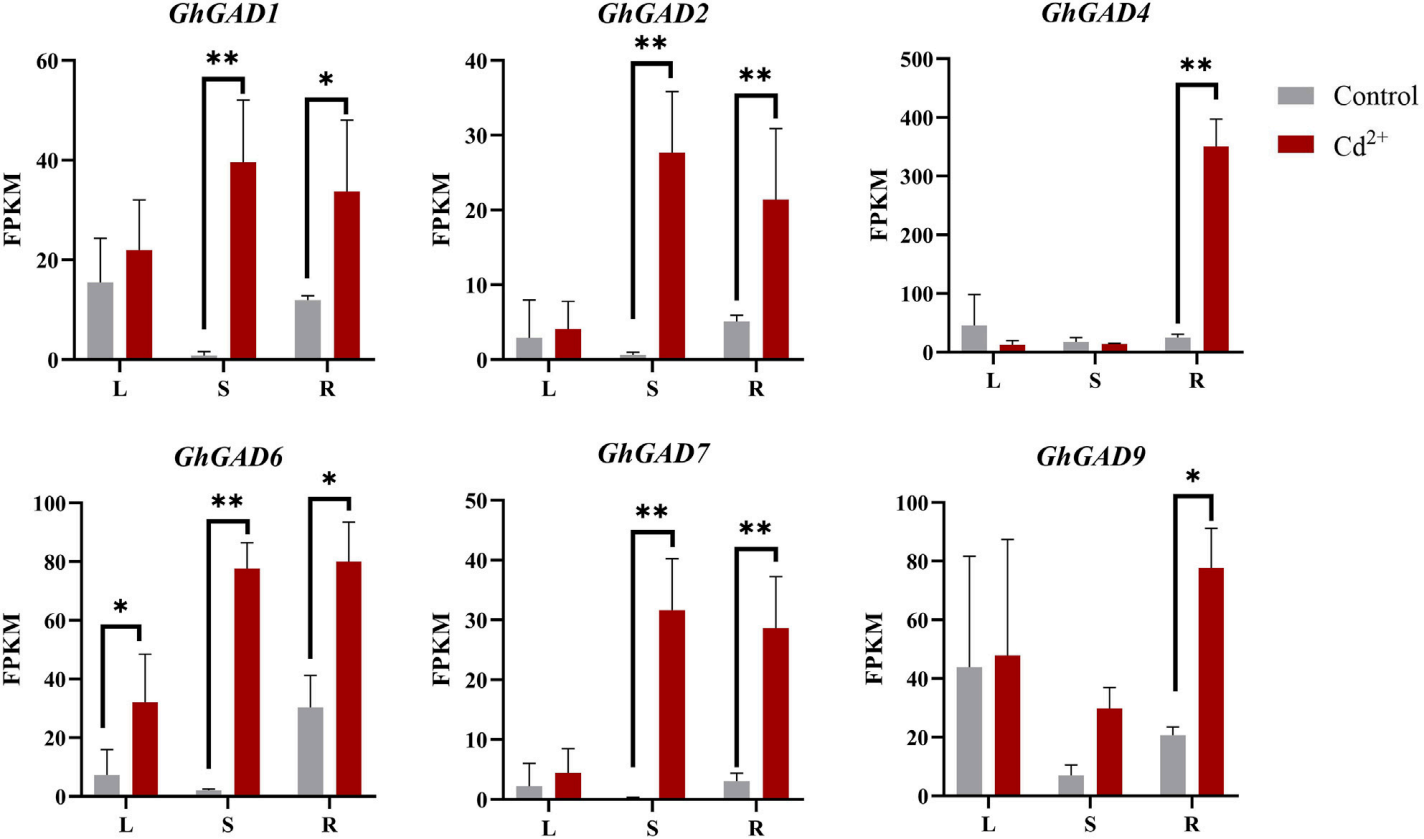
Expression analysis of clade Ⅲ GhGAD gene family members in different tissues under 4 mM Cd^2+^ treatment for 9 h. L, leaf; S, stem; R, root. *0.01 < *p* < 0.05, **p < 0.01.

The expression patterns of the *GhGADs* in clade Ⅲ were all up-regulated in roots under Cd^2+^ treatment (Figure 9). *GhGAD1*, *GhGAD2*, *GhGAD6,* and *GhGAD7* were significantly increased in the stem. Only *GhGAD6* responded to Cd^2+^ stress in roots, stems, and leaves. Therefore, we selected *GhGAD6* for further analysis.

### Interaction network of GhGAD6 protein

**FIGURE 10:**
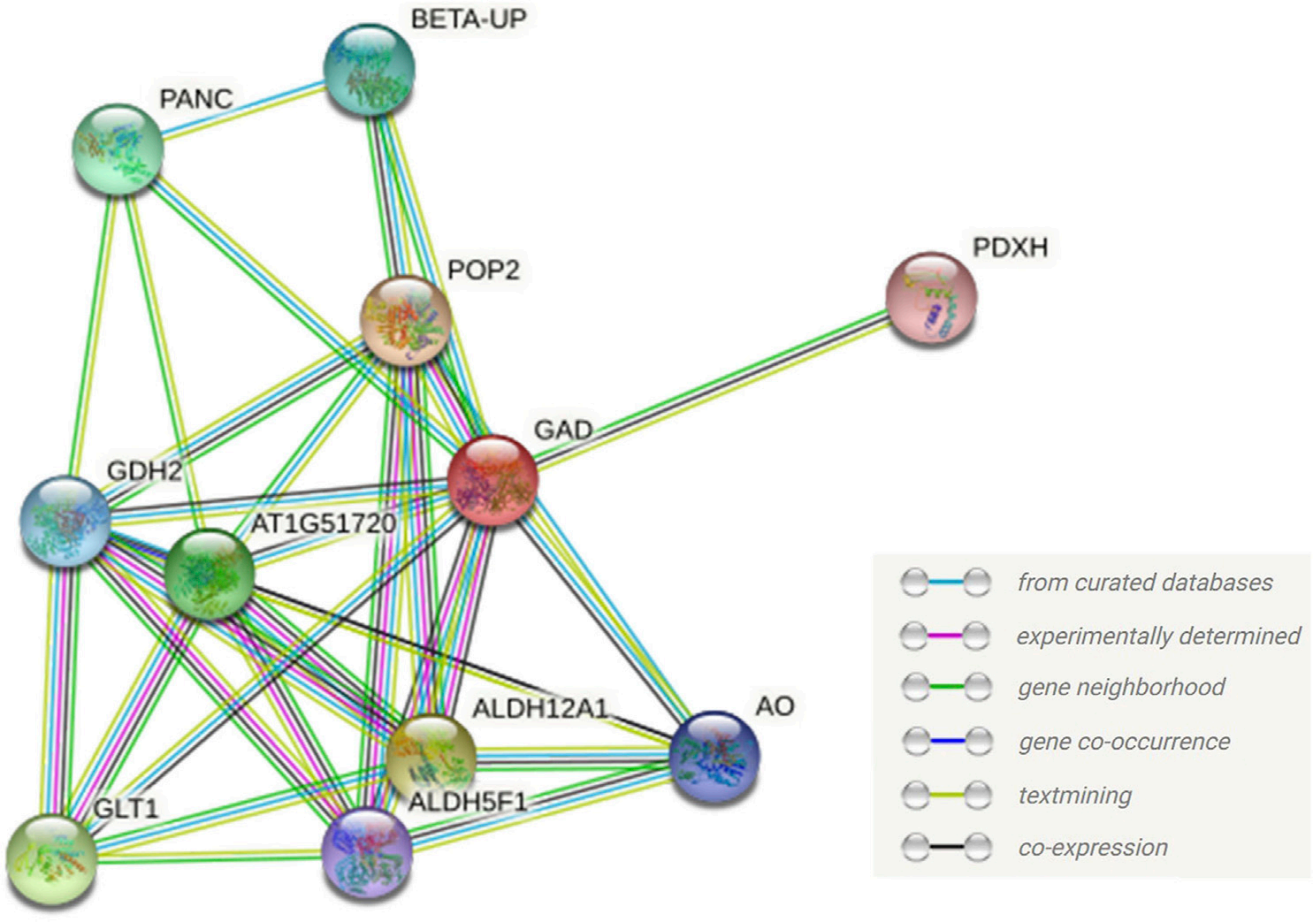
Interaction network of GAD protein. The GAD represented the protein AtGAD1 with the highest homology to GhGAD6. All proteins are *Arabidopsi*s proteins in the network.

Based on the homologous gene *ATGAD1* in *Arabidopsis* with the highest homology to GhGAD6, an interaction network was constructed using the STRING database to analyze the function of the GAD protein. (Figure 10). ATGAD1 interacts with 4-aminobutyrate pyruvate transaminase (POP2), succinate-semialdehyde dehydrogenase (ALDH5F1), delta1-pyrroline-5-carboxylate dehydrogenase (ALDH12A1), glutamate dehydrogenase (GDH), and glutamate synthase (GLT) proteins. By analyzing the KEGG pathway of GhGAD6 from the transcriptome data, we found that GhGAD6 was mainly involved in Alanine, aspartate, and glutamate metabolism (ko00250), GAD synthesized GABA through POP2, ALDH5F1 converted GABA to succinate, and then entered the TCA cycle. ALDH12A1, GLT, and GDH were involved in the synthesis of glutamate. We speculated that GAD interacted with these proteins to respond to Cd^2+^ stress by regulating the content of glutamate and GABA.

### Cotton plants with the *GhGAD6* gene silenced by VIGS were sensitive to Cd^2+^ stress

**FIGURE 11:**
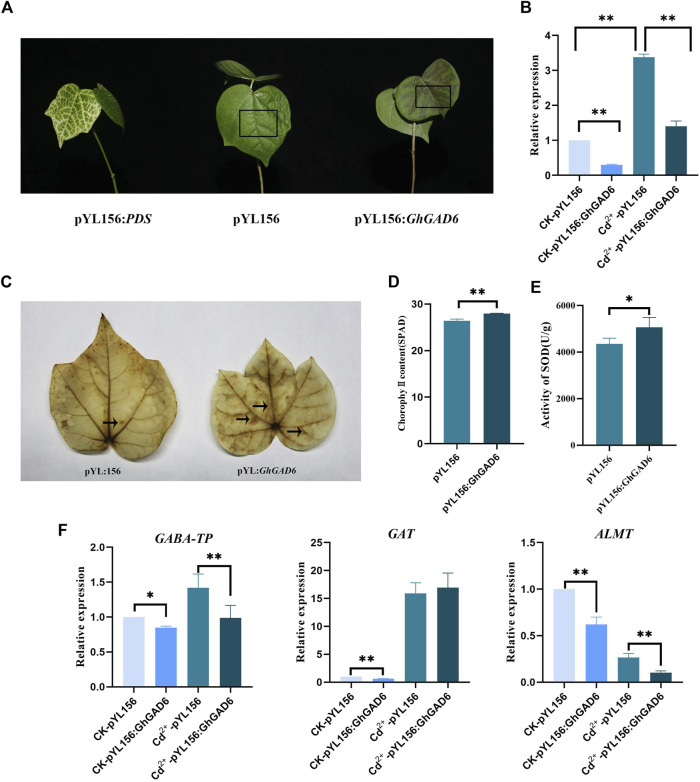
Silencing *GhGAD6* via VIGS increased sensitivity to Cd^2+^ stress. **(A)** The phenotype of cotton after *GhGAD6* gene silencing under Cd^2+^ stress. pYL156:*PDS* as a positive control, pYL156 was an empty vector as control, and pYL156:*GhGAD6* was *GhGAD6* silenced lines.**(B)** The relative expression level of *GhGAD6* under water and Cd^2+^ stress. **(C)** DAB staining. **(D)** ChlorophylⅡ content of empty control and VIGS plants under Cd^2+^ stress. **(E)** SOD activity of empty control and VIGS plants under Cd^2+^ stress. **(F)** Detection of gene expression of GABA shunt in GhGAD6 silenced lines. The black box represents the darkening of the leaf veins, the black arrows indicate the location of H_2_O_2_ generation.*0.01 < *p* < 0.05, **p < 0.01.

We used VIGS experiments to verify the role of *GhGAD6* under Cd^2+^ stress. The pYL156:*PDS* exhibited obvious chlorosis, and the relative expression level of *GhGAD6* was determined by qRT-PCR, which showed a 70% decrease in pYL156:*GhGAD6* than pYL156 (Figure 11B), indicating that it had a good silencing effect. After Cd^2+^ stress, cotton showed blackening of stems and veins, wilting of leaves, and pYL156:*GhGAD6* was more severe than pYL156 (Figure 11A). The DAB staining showed the same result, more brown was produced at the veins of pYL156:*GhGAD6* than pYL156, indicating that H_2_O_2_ was accumulated in pYL156:*GhGAD6* after Cd^2+^ stress (Figure 11C). It can be seen that both pYL156 and pYL156:*GhGAD6* are up-regulated of *GhGAD6* after Cd^2+^ stress, and the up-regulation range is 3–4 times, but pYL156:*GhGAD6* is still significantly lower than pYL156. Furthermore, the chlorophylⅡ and SOD content of pYL156:*GhGAD6* also increased significantly after Cd^2+^ stress (Figures 11D,E).

We detected the expression of GABA transaminase (*GABA-TP*), GABA transporter (*GAT*) in the GABA shunt, and GABA receptor protein aluminum-activated malate transporter (*ALMT*) in plants (Figure 11F). It was found that the expression of *GABA-TP*, *GAT,* and *ALMT9* decreased significantly after silencing *GhGAD6*, which indicated that the GABA content was reduced. After Cd^2+^ stress, the expressions of *GABA-TP* and *GAT* were up-regulated, and the up-regulated expression of GABA shunt genes coped with Cd^2+^ stress.

## Discussion

The type II PLP_deC enzymes were an important group of carboxylases among the PLP-dependent enzymes, including glutamate decarboxylases, serine decarboxylases, and aromatic amino acid decarboxylases. Aromatic amino acid decarboxylases included tyrosine decarboxylases, tryptophan decarboxylases, and Histidine decarboxylases, which evolved from the same common ancestor, using the same coenzymes and protein scaffold to catalyze the conversion of disparate amino acids (Sandmeier et al., 1994). Since the annotated genes were based on sequence homology, excessive sequence homology in the PLP_deC family can lead to misannotation of PLP_deC family members (Kumar, 2016). Some species' genome assembly quality was not high, there are long-term challenges for the annotation of the PLP_deC family. Key active site residues can be identified by optimizing bioinformatics methods to annotate PLP_deC family members, for example, a glycine was identified as a key residue in the TDC sequence in *Papaver somniferum*, and a serine occupied the same conserved motifs in the TYDC sequence (Torrens-Spence et al., 2014). Most GADs have a CaMBD domain and a conserved Trp catalytic site at the C-terminus, which can be used as a feature to identify the GAD family and other gene families from type II PLP_deC (Akama and Takaiwa, 2007).

With the development of genomics sequencing and genomics methods, it is possible to explore the function of the GAD family. In this study, a total of 29 GAD genes were identified in four *Gossypium*, and 38 GAD genes were identified in *A. thaliana*, *V. vinifera*, *P. trichocarpa*, *T. cacao*, *G. max*, *O. sativa,* and *Z. mays*. The evolutionary relationship, family expansion, selection pressure, and expression of the GAD family under different abiotic stress especially Cd^2+^ stress were analyzed, to provide important reference information for understanding the function of the GAD.

Diploid species such as *Arabidopsis*, *O. sativa,* and *Z. mays* had the same GAD family numbers as diploid species *G. raimondii*, and *G. arboreum*, consistent with previous reports (Shelp et al., 2012). The number of GADs identified in *G. max* and *P. trichocarpa* was inconsistent with the Genbank search, possibly due to the high sequence similarity of the PLP_deC family and biased genome annotation. GAD had undergone a massive expansion in higher plants (Kumar, 2016), and the number of GAD genes was not proportional to genome size. Gm*GAD5*, *GmGAD6*, *GmGAD7*, *VitGAD2*, *ZmGAD1*, *OsGAD2*, *OsGAD4* separate into clade Ⅳ. According to previous studies, *OsGAD2* lacks the CaMBD region at the C-terminus, and the C-terminus extends with an autoinhibitory domain (Akama and Takaiwa, 2007). According to the sequence alignment results, the C-terminal of the clade Ⅳ GAD lacked the conserved Trp residues and Lys cluster at the C-terminal (Supplementary Figure S1), so it was speculated that clade Ⅳ GAD does not bind CaM, and may be involved in a unique regulatory pathway through a novel Ca^2+^/CaM-independent pathway occurs. *Gossypium* and *T. cacao* GAD genes were closely related in the phylogenetic tree, proving that *Gossypium* and *T. cacao* had the same ancestor (Li et al., 2014).

Analysis of the domain of the GAD family, the PLP_deC domain is most from 16 to 445 of the protein sequence, the longest is *GhGAD8* and *GbGAD8*, from 14 to 468, the shortest is *GaGAD5*, from 23 to 419. GhGAD encoded 478 to 503 amino acids, therefore the PLP_deC domain accounts for more than 83% of the entire protein. Most of the sequences in the GAD family were highly conserved, and the diversity in the function of GAD family genes was mainly caused by the difference between the N terminal and C terminal sequences. Similarly, protein motifs in the phylogeny showed that the three clades of *Gossypium* GADs differ only in the N-terminal and C-terminal. Motifs were consistent in the same clade, indicating that the protein structure was highly conserved within a subgroup. The unique genes of different subgroups are highly conserved. The unique motifs of different subfamilies may be the reason for the different functions derived from GAD.

Gene structure determines the function of genes. Through the tissue-specific expression analysis, we found that clade Ⅰ GAD family (*GhGAD3* and *GhGAD8*) were only specifically expressed in stamens. There are deletions of motifs at both the N-terminal and C-terminal, which may affect gene expression. The expression levels of the clade Ⅱ GAD family (*GhGAD5* and *GhGAD10*) in each tissue were significantly higher than the other genes. In the intron-exon structure analysis, it was found that although the clade Ⅱ GAD family had the same number of introns as other genes, the length of the first intron was significantly longer than that of other genes. Introns encode snoRNAs, miRNAs, and enhancers, which regulate gene transcription and affect gene expression abundance (Chorev and Carmel, 2012). In *A. thaliana*, it was found that the first intron was a favorable position for intron enhancer, which was close to the transcription start site (TSS). Intron enhancers can coordinate with the promoter to regulate gene expression (Meng et al., 2021). We speculated that *GhGAD5* and *GhGAD10* had enhancers in the first intron to enhance their expression in various tissues, which may play an important role in plant growth and development.

WGD in plants is an adaptive mechanism to the environment. About 130 million years ago, the common ancestor of dicots underwent a genome-wide triploid event (Jaillon et al., 2007). Then, *Gossypium* underwent a WGD 60 million years ago, resulting in a 5-6-fold amplification of the genes. For tetraploid, the genome underwent at least a 30–36-fold genome doubling (Paterson et al., 2012). In this study, tetraploid cotton identified twice as many GADs as diploid cotton. The uneven distribution of GAD genes on each chromosome demonstrates the existence of genetic variation during evolution (Paterson et al., 2012). WGD played an important role in the evolution of GAD. Combined with the calculation of selection pressure, we found that environmental selection pressure was generally 0–0.49, indicating that cotton GAD genes tend to be conserved in the evolutionary process. Functional differences are limited after undergoing segmental duplication and WGD.

At present, GAD has been found to respond to a variety of adversity stresses and alleviate the stress damage in *Arabidopsis*, rice, maize, ginseng, and other species. Through *cis*-acting element analysis, GAD genes were found to be related to plant hormones (ABA, MeJA, GA, IAA, SA) and various stresses (low temperature, drought, hypoxia, defense, and stress responsiveness). The most abundant element in the GAD promoter is ARE, which is mainly associated with hypoxia induction. It is consistent with previous reports that GAD is up-regulated by hypoxia induction (Miyashita and Good, 2008; Mei et al., 2016). The expression levels of *GhGAD5* and *GhGAD10* increased at 1–6 h and decreased at 12 h after salt, heat, and drought treatments, which may be involved in the regulation of salt, heat, and drought. The expression levels of *GhGAD2* and *GhGAD7* were significantly up-regulated after cold stress, and the expression levels of *GhGAD4* and *GhGAD9* were up-regulated by PEG induction, indicating that the GAD gene family is involved in responding to various stresses.

Under Cd^2+^ stress, the clade Ⅲ GAD family members were significantly up-regulated, and the expression of GAD was up-regulated 7–41 times in the 3 h of stress under Cd^2+^ stress, indicating that the clade Ⅲ GAD family members responded to Cd^2+^ stress. The expression of *GhGAD6* was up-regulated in roots, stems, and leaves under Cd^2+^ stress. Cotton seedlings were more sensitive to Cd^2+^ stress after silencing the expression of *GhGAD6* and increased chlorophylⅡ content and SOD activity. Cotton reduced the photosynthetic rate and the activity of antioxidant enzymes under 5 mg·kg^–1^ Cd^2+^ stress (An et al., 2019). On the contrary, the content of chlorophylⅡ was decreased while the activity of antioxidant enzymes was increased under the treatment of 500 μM Cd^2+^ (Khan et al., 2013). It is speculated that it may be related to the treatment time and concentration of Cd^2+^. This study used a Cd^2+^ concentration of 4 mM, which belongs to high-concentration, which will reduce the activity of SOD after stress. However, when GAD was silenced, the expression of GABA shunt-related genes *ALMT9*, *GAT*, and *GABA-TP* were decreased, especially GABA receptor protein *ALMT9*, implying that *GhGAD6* silenced lines had decreased GABA content (Ramesh et al., 2015; Bown and Shelp, 2020).

When cotton was under Cd^2+^ stress, it will induce an increase in reactive oxygen species (ROS) (Khan et al., 2013; Chen et al., 2019; Han et al., 2019). Plants can rapidly respond to Cd^2+^ stress by scavenging ROS through antioxidant systems (PÉRez-Chaca et al., 2014). The increase in intracellular GABA content could alleviate oxidative damage, reducing the accumulation of Cd^2+^. Exogenous addition of GABA can also increase the content of GSH against Cd^2+^ stress (Seifikalhor et al., 2020; Zhao et al., 2020; He et al., 2021). The *GhGAD6* silenced lines were accumulated more ROS and more sensitive to Cd^2+^, It is presumed to be caused by the reduction of GABA content in *GhGAD6* silenced lines. GAD is a key gene for GABA synthesis. The up-regulation expression of GAD can increase the content of GABA, and alleviate the Cd^2+^ poisoning by alleviating the oxidative damage caused by Cd^2+^ stress. Silencing of *GhGAD6* reduced GABA levels resulting in the accumulation of ROS, and cotton suffered severe Cd^2+^ toxicity.

## Conclusion

In this study, GAD was comprehensively identified for the first time in the four *Gossypium*, 10, 9, 5, and 5 GAD genes were identified in *G. hirsutum*, *G. barbadense*, *G. arboreum*, and *G. raimondii*, respectively. GAD was divided into four clades based on a phylogenetic tree, gene structure, and motifs composition. The segmental duplication was the main way of the GAD gene family evolutionary. Expression patterns analysis found that Clade Ⅲ GAD was induced by Cd^2+^ stress, especially *GhGAD6*. Silencing *GhGAD6* will lead to more severe Cd^2+^ poisoning in cotton, indicating that *GhGAD6* is involved in the response to Cd^2+^ stress in cotton. This study provides a reference for further exploring the function of GhGAD and Cd^2+^ stress.

## Data Availability

The datasets presented in this study can be found in online repositories. The names of the repository/repositories and accession number(s) can be found in the article/Supplementary Material.
