# Supplementary material for: Genome-wide identification of GAD family genes suggests GhGAD6 functionally respond to Cd2+ stress in cotton
Source: Front Genet. 2022 Sep 13;13:965058. doi: 10.3389/fgene.2022.965058 (PMC9513066; doi:10.3389/fgene.2022.965058)
Supplement: Supplementary file 1 [file Presentation1.zip › Suppl. Figure 1.DOCX]

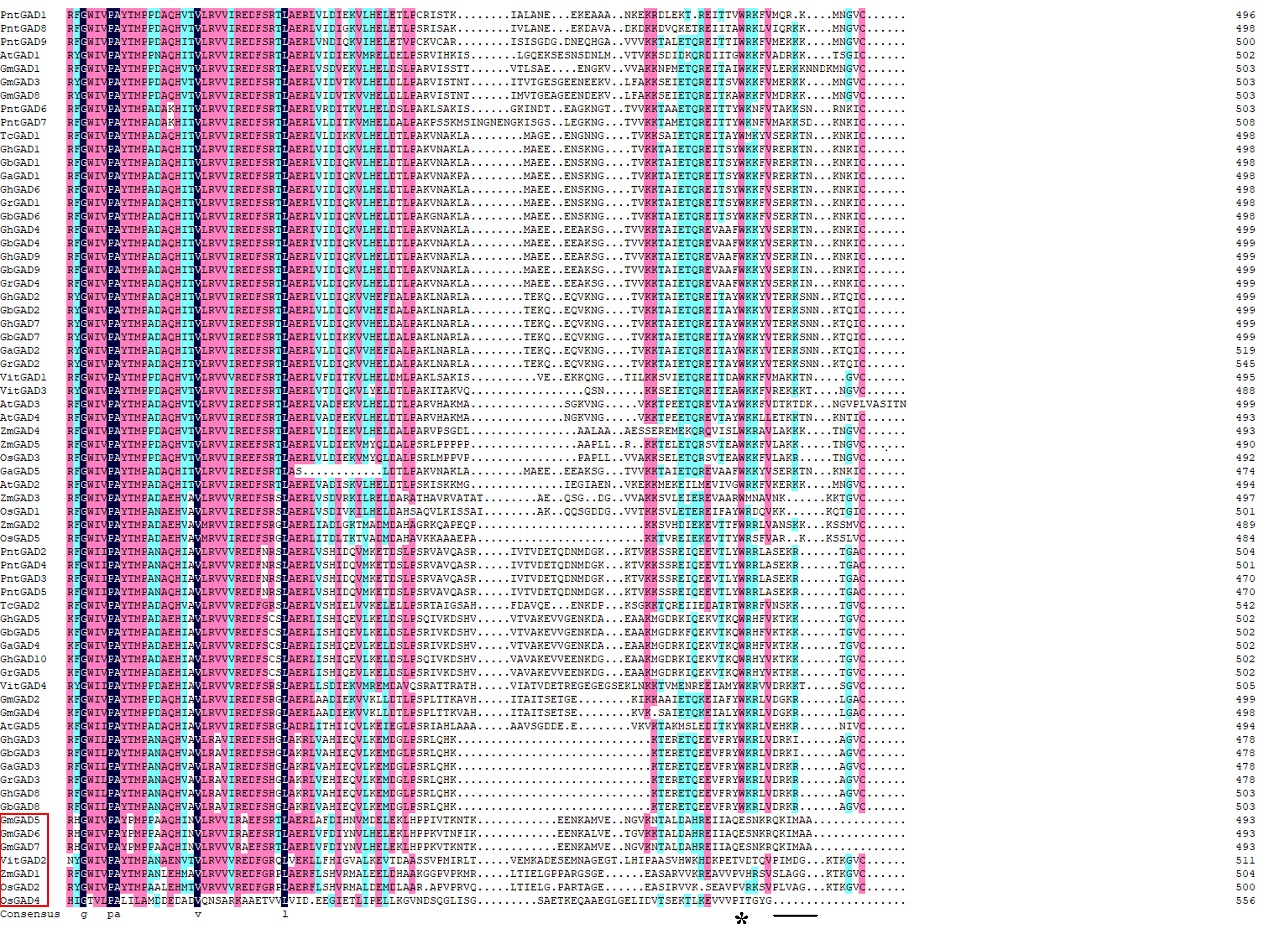


**Fig.S1.** Multiple sequence alignment of the C-terminal segments of plant GADs. Comparison of the C-terminal amino acid sequences of 67 identified GAD genes from *G. hirsutum*, *G. barbadense*, *G. arboretum*, *G.raimondii*, *A. thaliana*, *V. vinifera*, *P. trichocarpa*, *T. cacao*, *G. max*, *O. sativa* and *Z. mays*. Identical and similar amino acids are indicated by black and red. The red box represents the clade Ⅳ of GAD family. Trp (W) and the cluster of Lys (K) present in the C-proximal region are indicated by asterisks and a thick line, respectively.
